# Development and research of a SS-level universal double-beam assembled bridge barrier

**DOI:** 10.1038/s41598-024-55587-4

**Published:** 2024-02-27

**Authors:** Qianmiao Bu, Xinpeng Ning, Wen Zhang, Lei Ma

**Affiliations:** 1 Rioh Traffic Safety Co., Ltd., Beijing, 100088 China; 2 Xinjiang Academy of Transportation Sciences Co., Ltd., Ürümqi, 830000 China; 3 Key Laboratory of Highway Engineering Technology in Arid & Desert Region, Ministry of Transport, Ürümqi, 830000 China

**Keywords:** Worst-case bridge barrier base, Bridge barrier safety improvement, Finite element simulation, Yield line theoretical calculations, Real-vehicle impact test, Composites, Computer science, Computational science

## Abstract

A wide variety of bridge barriers are used on highways. The bearing capacities of different types of deck slabs are measured in this study by applying the yield line theory to determine the worst-case scenario. An improved configuration for the worst-case barrier base and deck slab is developed, namely the universal double-beam assembled bridge barrier, which can enhance the safety performance of exiting concrete-base bridge barriers. According to the simulated impact test results, the new barrier meets SS-level requirements in terms of containment, redirective, and buffering performance as specified in the *Standard for Safety Performance Evaluation of Highway Barriers* (JTG B05-01-2013). The barrier structure’s compatibility with various bases is also analyzed. SS-level impact tests are conducted on real vehicles, including cars, buss, and trucks. The results show that the safety performance of the new barrier configuration reaches SS-level, and the barrier is universally compatible with concrete bases with a height of 63 cm and above.

## Introduction

In recent years, the number of highway bridges in China has been increasing straight-line, reaching 912,800 in 2020. There are different types of safety facilities for bridge deck systems with varied protection performance, depending on the construction year, level, and standard.

Before 1994, there was no specific design specification for barriers in China. The second paragraph of Article 10.0.1 of *Highway Engineering Standard* (JTJ 01-88) states that “barriers should be installed on bridges of expressways and level-1 highways.” From 1994 to 2006, bridge barriers were designed with reference to expressway barriers, represented by an assembled PL_2_-level bridge barrier with a protection energy of 130 kJ^1^. In 2006, China issued the *Guidelines for Design of Highway Safety Facilities* (JTG D81-2006)^2^, which stipulates that the protection level of a highway bridge barrier should at least reach SB-level with a protection energy of 280 kJ. When renovating or expanding bridge barriers, the protection level shall be determined according to the *Design Specifications for Highway Safety Facilities* (JTG D81-2017)^3^.

Bridges constructed before 2006 have been in use for more than 15 years. Although the bridge barriers meet the standards at the time they were built, the vehicles and traffic flow characteristics have changed, and the carrying capacity and load of vehicles have increased with the continuous development of economy and society. The public is now expecting higher standards for the safety protection performance of highway bridges^4^. We found through field survey that there may be various types of bridge barriers on the same expressway, such as F-shaped concrete barriers, single-slope concrete barriers, assembled barriers, and corrugated beam barriers^5^. The situation is more complicated for expressways built before 1994—even if one section is only equipped with assembled barriers, the height of the concrete base, the angle of the impact face’s gradient change point, and the diameter of the cross beam may still be different.

In projects that aim to improve the safety level of bridge barriers, complete demolition and reconstruction will inevitably cause large amounts of waste, high investment, and a long construction period, severely affecting highway operations. However, if we want to keep the existing barriers while making certain improvements, there are also many challenges. For example, the barriers and deck slabs may vary in the structure and shape, and the reinforcement and height may be different. It is unpractical to tailor-make an improvement configuration for each type of barriers. Thus, such improvement projects should be planned in a systematic manner, and local conditions must be fully considered^4^.

The combination of finite element calculation and actual vehicle collision tests is an effective method for studying the protective capacity of guardrails^6^. Ray et al., summarized the design and analysis of the Annisquam River Bridge railing to satisfy the requirements of National Cooperative Highway Research Program (NCHRP) Report 350^7^ Test Level 3. Design of a previously accepted Minnesota Test Level 3 bridge railing was used as the starting point. A baseline finite-element model of a crash-tested Minnesota bridge railing is developed and validated against the full-scale crash test results, using the non-linear dynamic finite-element program LS-DYNA^8^. Yao et al., presented a novel style of assembled rolling guardrail, and a vehicle-guardrail collision numerical model is built in LS-DYNA. The performance of Beam-column guardrail (B-C.G), Assembled Guardrail (A.G), and Assembled rolling guardrail (A-R.G) is evaluated^9^.

In this paper, the worst-case barrier base and bridge deck of a highway are studied, and a universal improvement scheme is developed. The novelty of this article lies in the investigation and analysis of existing bridge guardrail bases and bridge decks. The yield line theory is used to calculate the bearing capacity of different bridge guardrail bases and bridge decks, and the most unfavorable concrete bases and bridge decks are selected. Based on this, the bridge guardrail structure design method is used to carry out the improvement plan design, and the protection ability of the improvement plan is verified through finite element simulation and full-scale vehicle collision tests.

## Current bridge barrier bases

There are three types of bridge barriers on the discussed highway, which are assembled barrier, concrete barrier, and corrugated beam barrier. Through a comparative study of the design drawings of the barriers and a field survey, the worst-case concrete base is determined.

**Figure 1:**
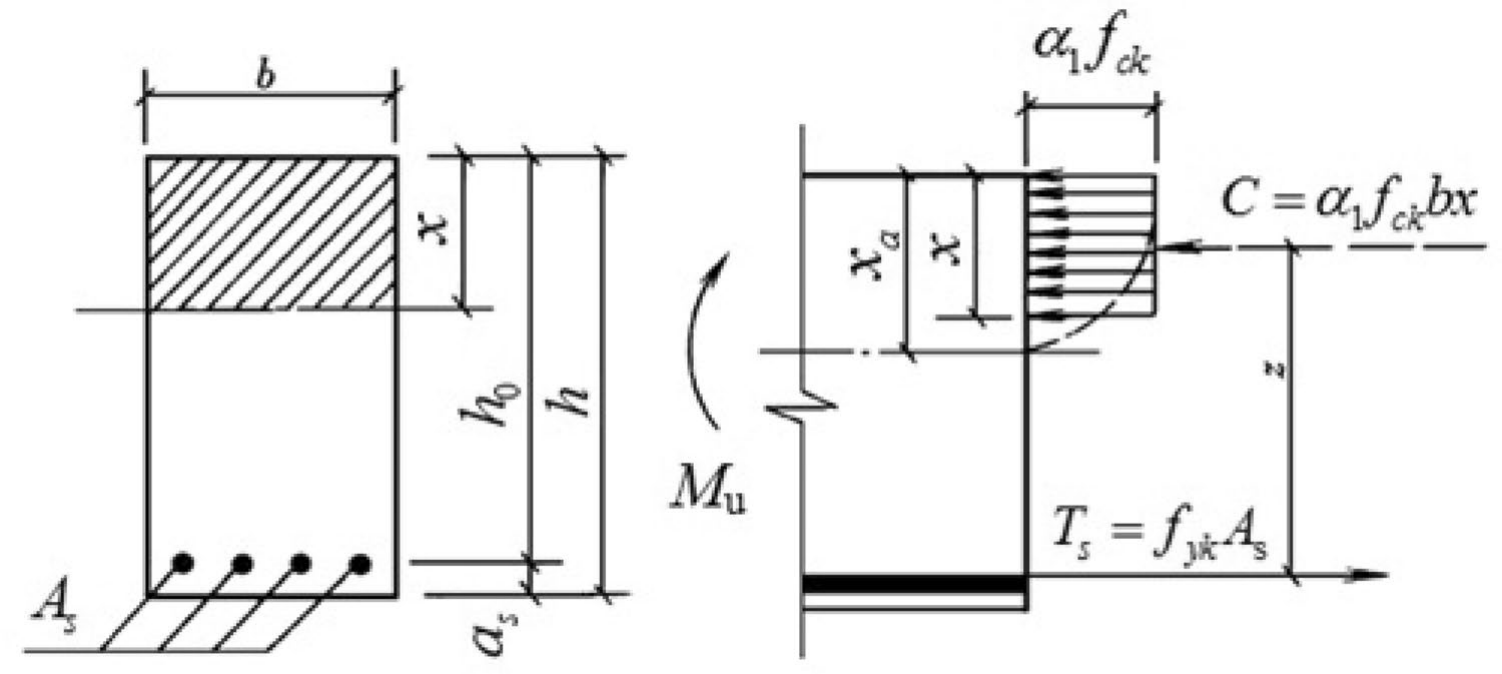
Rectangular section with single-steel reinforcement.

According to the Appendix D of the *Design Specifications for Highway Safety Facilities* (JTG/T D81-2017)^10^, the bearing capacity of concrete barriers mainly comes from reinforcement and concrete, based on theoretical calculations. The theoretical calculation method was introduced in the *Technical Guide for Enhancing Highway and Bridge Safety Performance*^4^ as a suitable approach for measuring the bearing capacity of reinforced concrete flexural members with a single-steel rectangular section, as shown in Fig. 1.

Flexural capacity of the concrete base of the bridge barrier:

1 $$M = A_{s} f_{yk} \left( {h_{0} - \frac{x}{2}} \right)$$

*M*—the standard value of bending moment generated by the load on this section; $A_{s}$—the cross-sectional area of longitudinally stressed steel bars in the tensile zone; $f_{yk}$—the standard value of yield strength of steel bars; $h_{0}$—the effective height of the cross-section; $x$—the height of the compression zone calculated according to the equivalent rectangular stress graph.

**Figure 2:**
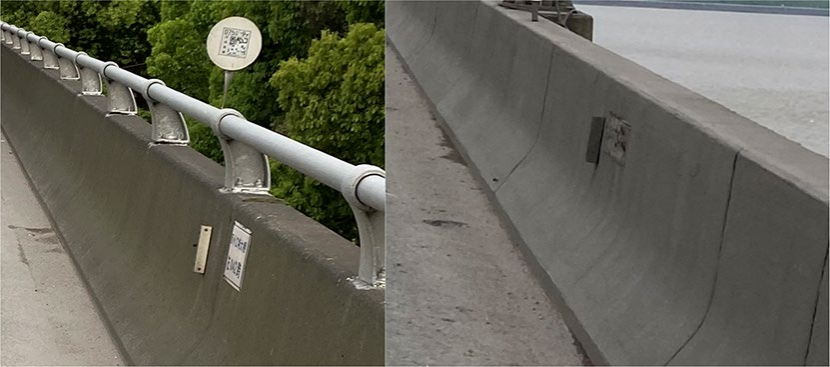
Concrete base with a height of 81 cm.

**Figure 3:**
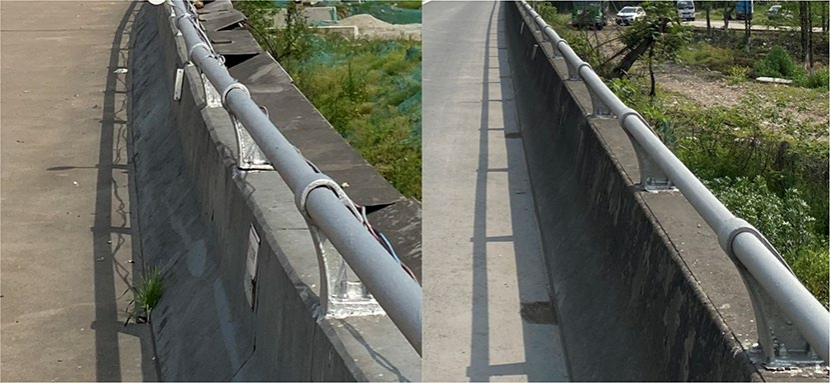
Concrete bases with heights of 63 cm and 68.5 cm.

**Figure 4:**
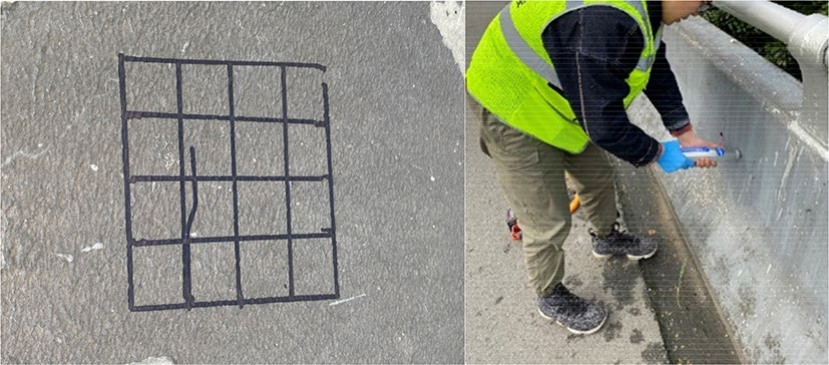
Detection of concrete strength using rebound method.

**Figure 5:**
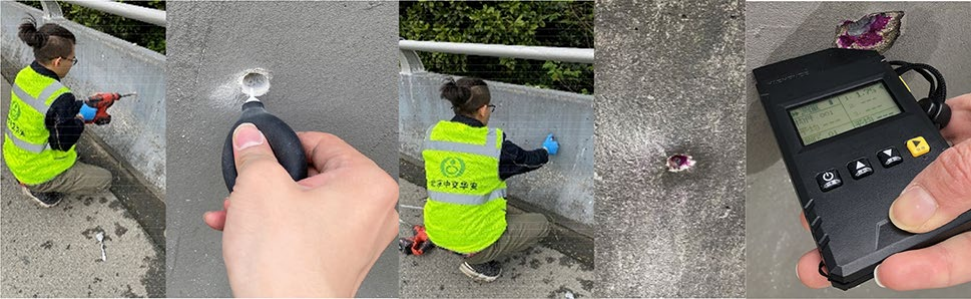
Detecting the depth of concrete carbonation.

The analysis shows that the bearing capacity of assembled barrier and concrete barrier is directly related to the height of concrete when there is little difference in reinforcement. The height of the concrete bases of the three types of bridge barriers on the studied highway is 81 cm, 63 cm, and 68.5 cm, respectively, as illustrated in Figs. 2 and 3. After using the rebound method to test the concrete strength and carbonization depth, the estimated minimum strength value of the guardrail base concrete is 18 MPa after conversion, as shown in Figs. 4 and 5. The reinforcement effect of a barrier is directly proportional to its height. Generally, the reinforcement area is larger for higher barriers. Thus, according to the equation, the shortest base can be regarded as the worst case, i.e. the concrete base measuring 63 cm in height.

## Current deck slabs

A site inspection and review of the design drawings reveal that the deck slabs using simply supported T-beams are at least 10 cm thick. According to the calculation method for the bearing capacity of bridge decks in Appendix D of the *Design Specifications for Highway Safety Facilities* (JTG/T D81-2017)^10^, when a large vehicle collides with the existing bridge barriers, the barriers will bear the impact force and transmit the collision moment to the deck slab, which will greatly impact the overall safety performance of the barriers while causing irreparable damage to the bridge.

**Figure 6:**
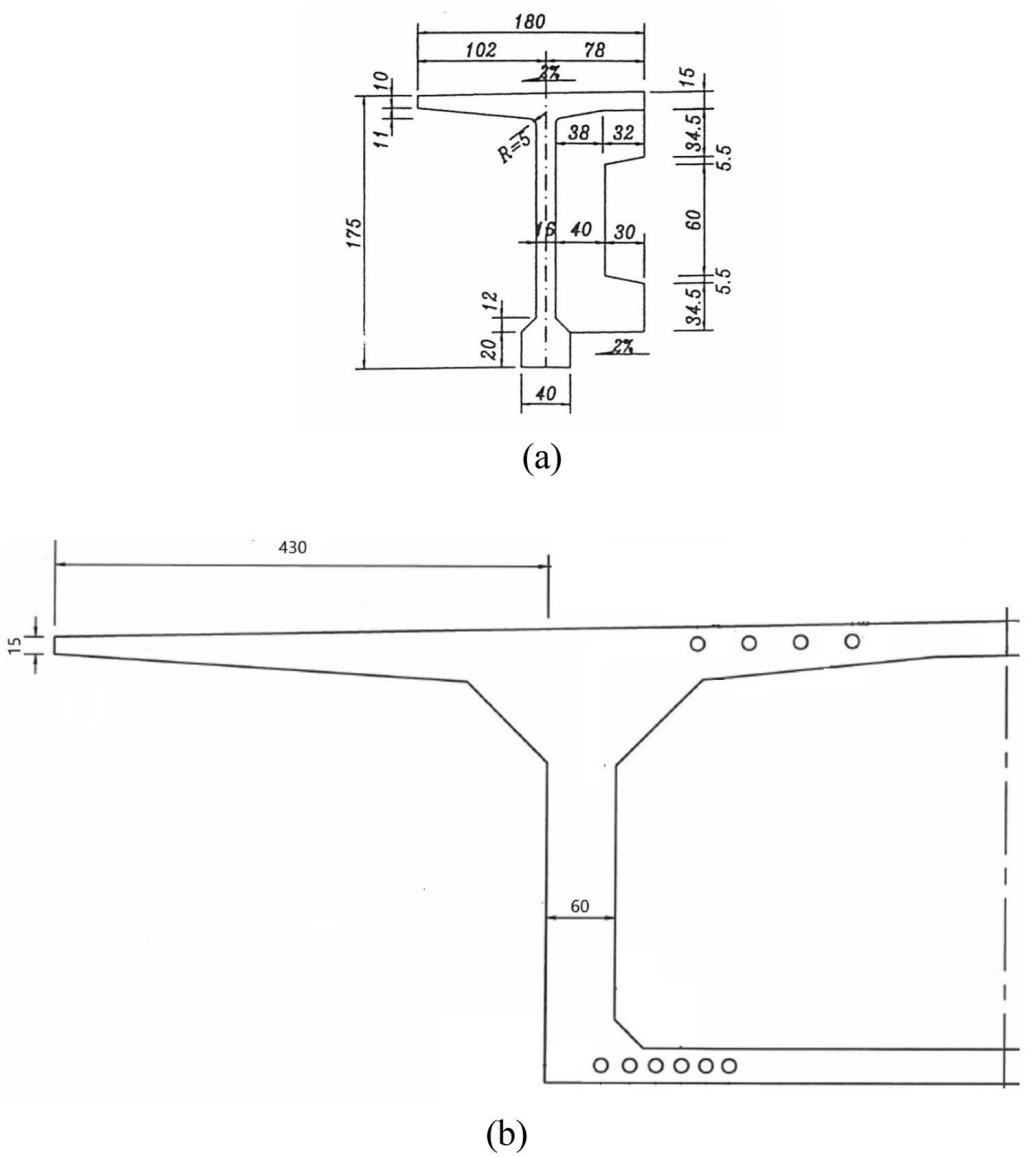
T-beam and box girder (cm).

By investigating various types of bridge decks of the highway, the one with the thinnest edge and the longest cantilever length is selected, as shown in Fig. 6.

According to the theoretical calculation method of yield line recommended by JTG/T D 81-2017^10^, the bearing capacity of the bridge deck was calculated, and the weakest carrying capacity case is the T-beam bridge deck with an edge thickness of 10 cm and a cantilever length of 94 cm (Table 1).

**Table 1:** Bearing capacity of deck slabs on an expressway bridge.

S/N	Type of bridge deck	Edge thickness (cm)	Cantilever length (cm)	Bearing capacity in state I kN m/m	Bearing capacity in state II kN m/m
1	T-beam	10	94	6.74	10.6
2	Box girder	15	430	38	47.6

## Design of the universally applicable improved configuration

**Figure 7:**
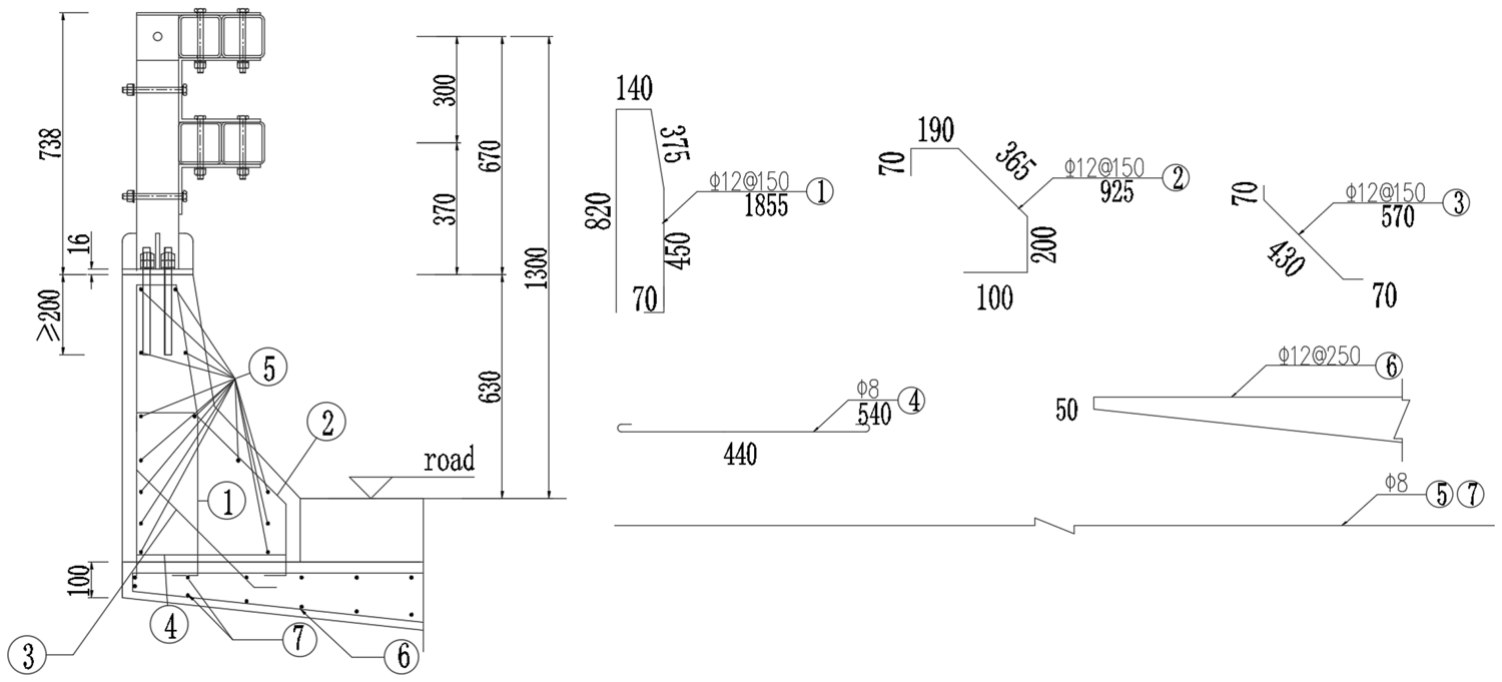
Cross-sectional view of the universal double-beam assembled bridge barrier.

Following the review of current bridge barrier bases and deck slabs, an improved configuration was developed based on the current barrier with a 63 cm-high concrete base and a T-beam bridge deck with a thickness of 10 cm and a cantilever length of 94 cm. The improved configuration has removed the upper beam and added a new beam column, forming an assembled barrier with a column spacing of 2 m, as shown in Fig. 7. The ①②③ steel bars are the hoops of the barrier, with a spacing of 150 mm. The ④ steel bar connects the ① and ② steel bars together. The ⑥ steel bars are the hoops of the T-beam bridge deck, with a spacing of 150 mm. The ⑤ and ⑦ steel bars are the longitudinal bars of the guardrail and bridge deck, respectively. The diameter of the ①②③⑥ steel bar is 12 mm, and the diameter of the ④⑤⑦ steel bar is 8 mm. The tensile strength of the ①②③⑥ steel bar is 335 MPa, and the tensile strength of the ④⑤⑦ steel bar is 335 MPa.

The *Design Specifications for Highway Safety Facilities* (JTG D81-2017) put forward construction requirements for bridge barriers^3^. The distances between the center of the upper beam and the bridge deck, and between the center of the lower beam and the bridge deck are 1300 and 1000 mm, respectively. The beams are all made of the same material. The resistance ratio between the beams is the same as their modulus ratio. The weighted average height $\overline{Y}$ of the load-bearing beams from the bridge deck can be calculated as the weighted average height of each beam’s center. The total height meets the requirement that the height of a SS-level assembled bridge barrier should be at least 120 cm.

2 $$\overline{Y} = \frac{{\sum \left( {RiYi} \right)}}{{\overline{R}}} = \frac{1300 + 1000}{2} = 1150 \;\;{\text{mm}}$$

By adding spacer blocks between the upright posts and cross beams, the upright posts’ back distance is increased to 300 mm and the clearance between the cross beams increased to 180 mm, thereby reducing the possibility of the upright posts being directly impacted by wheels, bumpers, or engine covers. The total height of the beam in contact with the vehicle is 240 mm, and its ratio to the height of the column $\frac{\sum A}{H} = \frac{240}{{670}} = 0.35$. The back distance of the upright posts complies with relevant standards.

The splicing sleeve of the beam is made of the same material as the beam, with a length of 500 mm, more than twice as wide as the beam. The connecting bolts are 10.9S bolts.

The design of the universal double-beam assembled bridge barrier complies with the *Design Specifications for Highway Safety Facilities* (JTG/T D81-2017)^10^.

## Safety performance evaluation indicators

The *Standard for Safety Performance Evaluation of Highway Barriers* (JTG B05-01-2013) specifies the evaluation indicators of barriers as well as experimental conditions for real-vehicle impact tests^11^.

**Figure 8:**
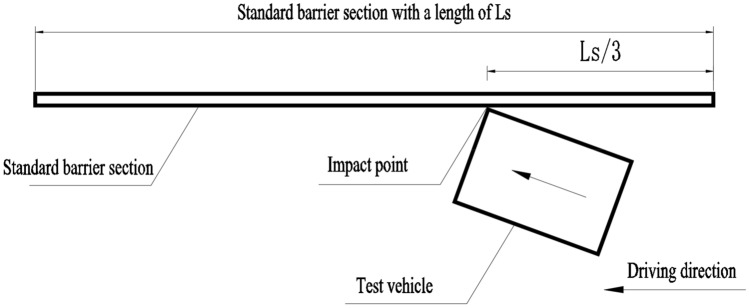
Impact point on the standard barrier section.

During the verification tests of the improved bridge barrier configuration, the impact point for the small and buss and the truck shall be at 1/3 of the distance from the starting point of the standard barrier section along the driving direction. The setup of the tested barrier and the impact point is illustrated in Fig. 8.

Containment, redirective and buffering performance of the barrier are measured and analyzed based on the results of the impact tests.

Containment refers to that the barrier shall be capable of containing the vehicle from crossing through, climbing over, or riding over it after an impact. Components of the tested barrier and their breakaway parts shall not penetrate the vehicle’s passenger compartment.

Buffering requires that the occupant impact velocity shall not exceed 12 m/s both longitudinally and transversely. In no case shall the longitudinal or transverse component of the post-impact acceleration exceed 200 m/s^2^.

The collision speed of passengers is calculated according to Eq. (3):

3 $$v_{x,y} = \mathop \smallint \limits_{0}^{{t^{*} }} a_{x,y} dt$$

$v_{x,y}$—passenger collision velocities in longitudinal (x-direction) and transverse (y-direction) directions; $a_{x,y}$—acceleration at the center of gravity of the vehicle in the longitudinal (x-direction) and transverse (y-direction) directions; $t^{*}$—the time when the hypothetical passenger's head collides with the interior of the passenger compartment is taken as the time when the hypothetical passenger’s head moves 0.6 m longitudinally (x direction) or 0.3 m laterally (y direction) inside the passenger compartment, calculated according to Eq. (4):

4 $$X,Y = \mathop \smallint \limits_{0}^{{t^{*} }} \mathop \smallint \limits_{0}^{{t^{*} }} a_{x,y} dt$$

X = 0.6 m, Y = 0.3 m, $t^{*}$ is the smaller value of $t_{x}^{*}$ and $t_{y}^{*}$ obtained by satisfying the integration equations in the x and y directions.

According to the acceleration data of the collision test vehicle's center of gravity, t* can be calculated from Eq. (4), and then the longitudinal and transverse components of the passenger collision velocity can be calculated from Eq. (3). The absolute value should be less than or equal to 12 m/s. During the process of vehicle collision with the guardrail, after the hypothetical collision between the passenger’s head and the interior of the passenger compartment, the longitudinal and transverse components of the acceleration at the center of gravity of the vehicle can be calculated at intervals of 10 ms. The maximum absolute value should be less than or equal to 200 m/s^2^.

Redirecting refers to that the vehicle shall not roll over after an impact, and the wheel marks of the vehicle after leaving the exit point shall meet the requirements for redirective exit box.

**Figure 9:**
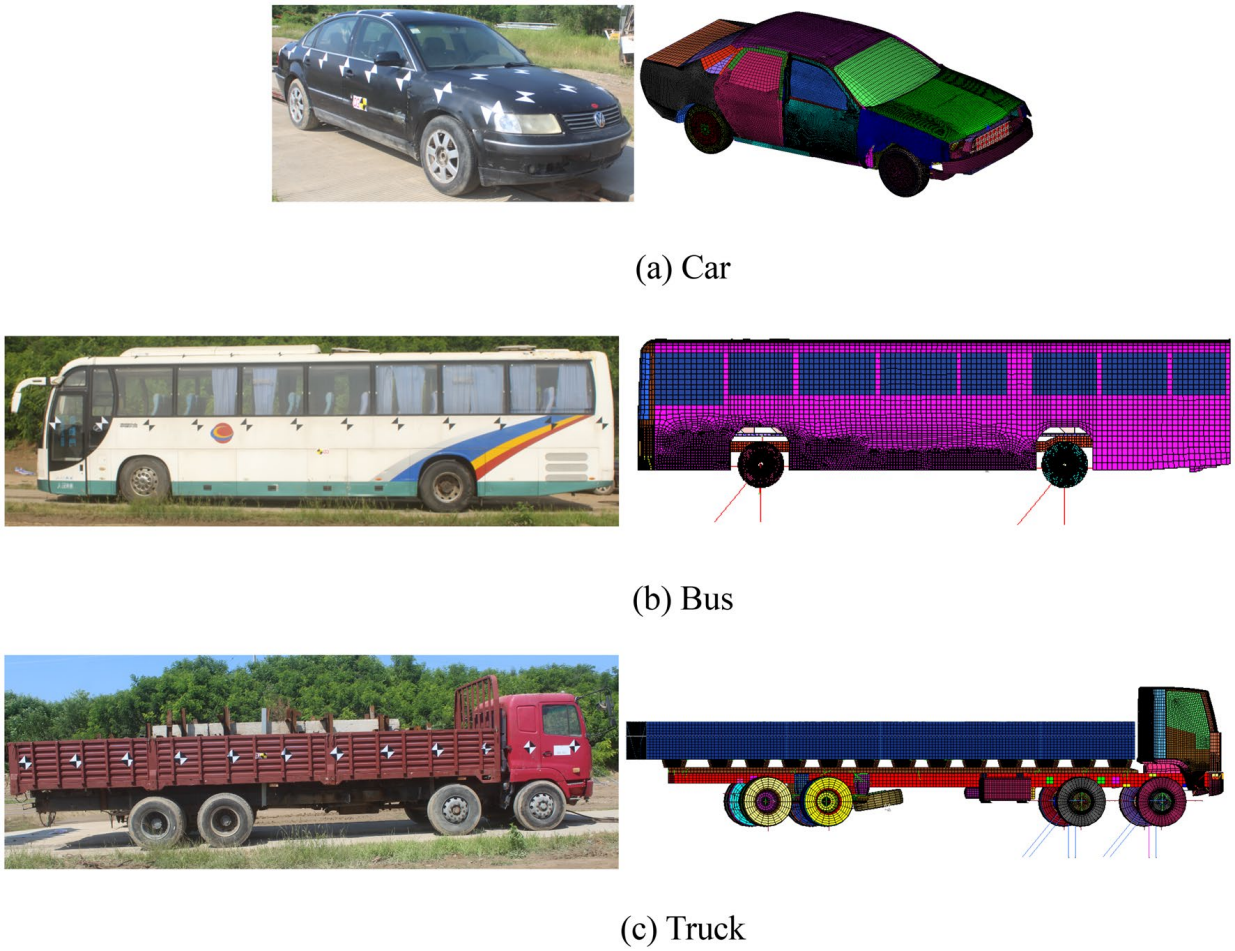
Real-vehicle and numerical-vehicle.

Table 2 displays the test conditions for SS-level barriers of the numerical model, the real-vehicle and JTG B05-01-2013^11^. Real-vehicle and numerical-vehicle are shown in Fig. 9.

**Table 2:** SS-level impact conditions.

Safety level	Vehicle type	Numerical-vehicle			Real-vehicle			JTG B05-01-2013 requirements		
		Total mass of the vehicle (t)	Impact velocity (km/h)	Impact angle (°)	Total mass of the vehicle (t)	Impact velocity (km/h)	Impact angle (°)	Total mass of the vehicle (t)	Impact velocity (km/h)	Impact angle (°)
Level VI (SS)	Car	1.51	100	20	1.46	100.11	20.3	1.5	100	20
	Bus	18.09	80	20	18.09	80.33	20.4	18	80	20
	Truck	33.11	60	20	33.27	60.54	20.1	33	60	20

**Figure 10:**
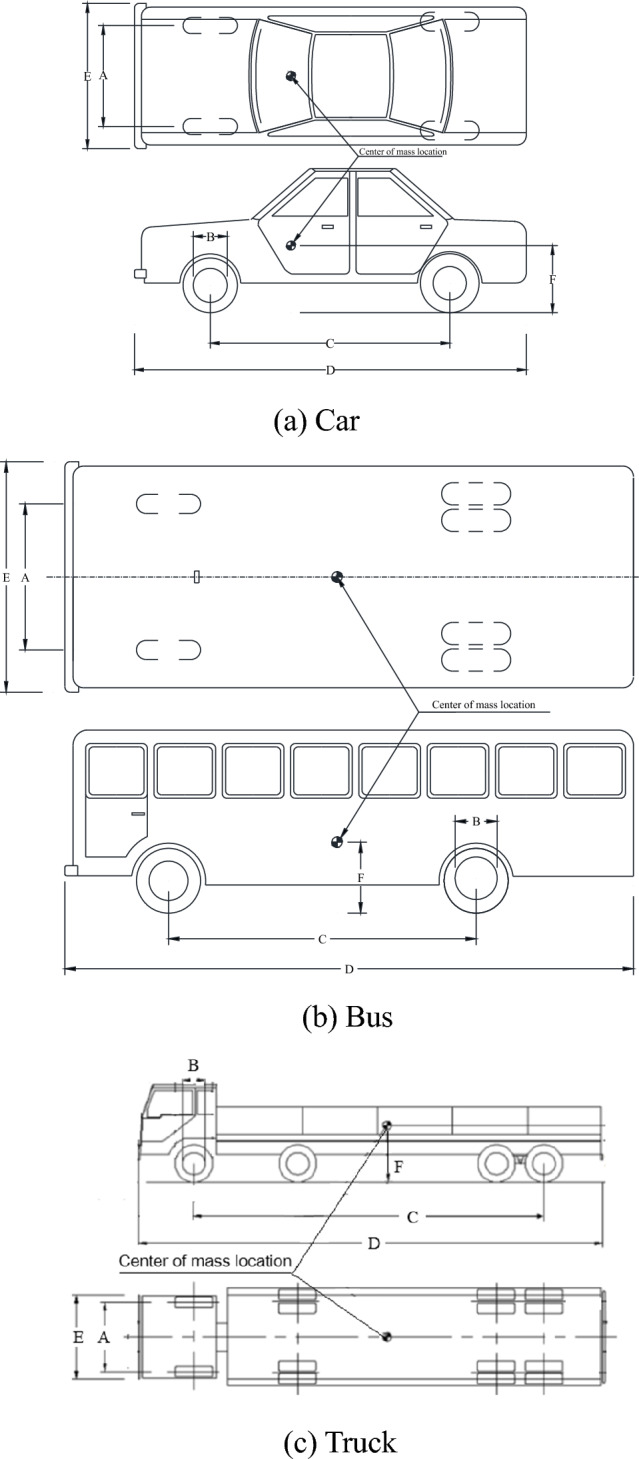
Vehicle technical parameters.

Table 3 displays the vehicle technical parameters for SS-level barriers of the numerical model, the real-vehicle and JTG B05-01-2013^11^. The vehicle technical parameters is shown in Fig. 10.

**Table 3:** Vehicle technical parameters.

Safety level	Vehicle type	Numerical model (mm)						Vehicle (mm)						JTG B05-01-2013 requirements (mm)					
		A	B	C	D	E	F	A	B	C	D	E	F	A	B	C	D	E	F
Level VI (SS)	Car	1500	415	2728	4600	1780	530	1500	440	2800	4610	1560	550	1500	320	2610	4600	1770	580
	Bus	2100	477	6505	12,687	2551	1364	2030	600	6100	11,800	2450	1180	2050	520	6010	11,910	2520	1290
	Truck	2034	486	8520	12,577	2486	1723	2010	600	7820	11,970	2200	1730	1950	520	7610	11,900	2490	1910

Note: A—front track; B—wheel radius; C—wheelbase; D—total length; E—overall width; F—centroid height.

## Safety performance analysis

### Establishment of the numerical and the full-scale barrier

**Figure 11:**
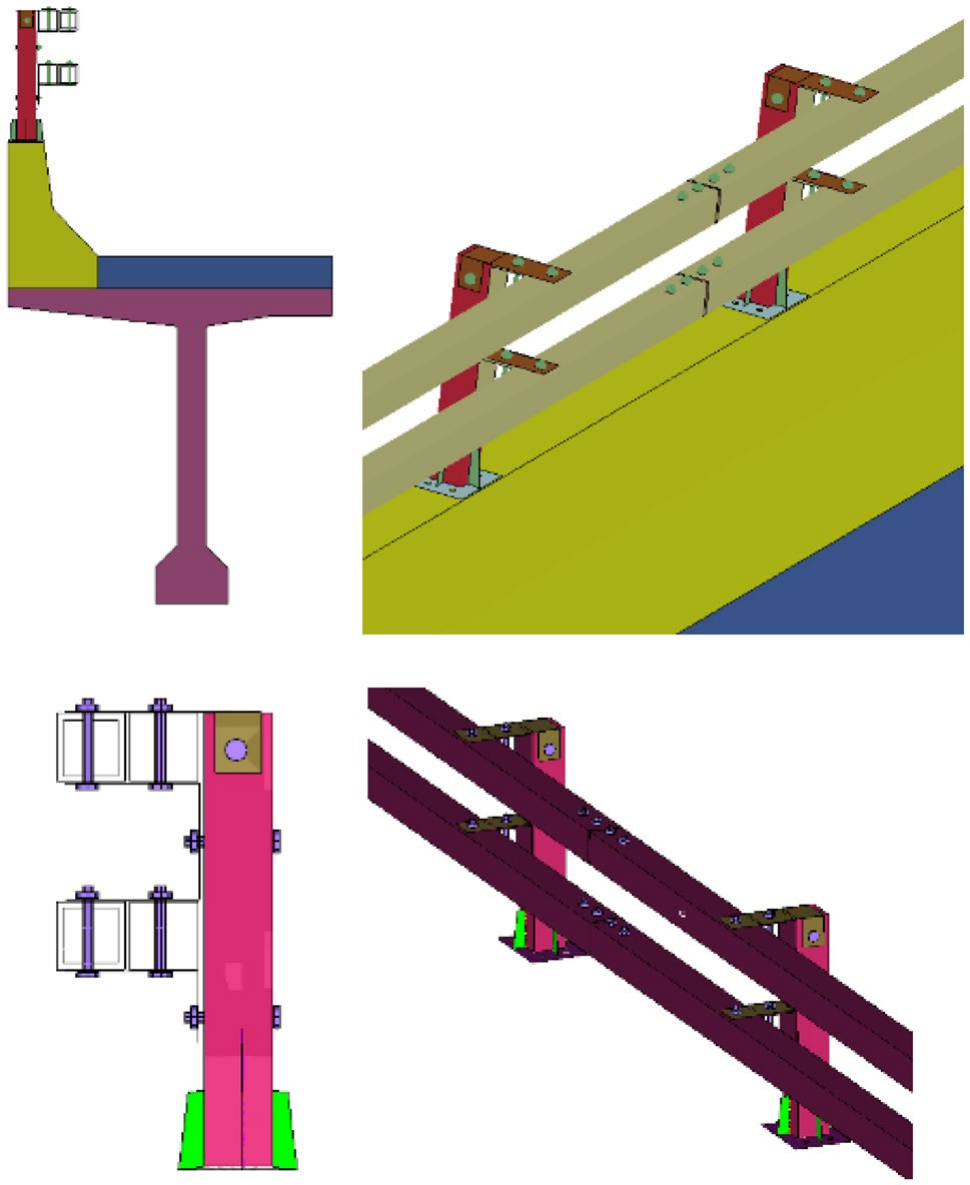
Finite element model of the universal double-beam assembled bridge barrier.

The universal double-row beams and upright posts are made of isotropic Q355. Having been applied external force of 355 MPa which is yield strength, the material enters a plastic stage until it is stretched and broken. Parameters of the material are defined by using the lsdyna keyword *MAT_PIECEWISE_LINEAR_PLASTICITY, with a density of 7.85 kg/m^3^, Poisson’s ratio of 0.3, elastic modulus of 210 GPa and yield strength of 355 MPa. Section characteristics are defined by using the lsdyna keyword *SECTION_SHELL. Beams, upright posts, and spacer blocks are set as Belytschko-Tsay shell units with a thickness of 6 mm. The concrete model is selected for both the barrier and deck slab, which are defined by using the keyword *MAT_CSCM_CONCRETE. The barrier concrete is 18 MPa in terms of strength grade, and the deck concrete is C40, which standard value of axial compressive strength is 26.8 MPa. The characteristics of the cross-section are set as entity units by using *SECTION_SOLID. The reinforcement is simulated as a line element by using *SECTION_BEAM, and the material is Q235 steel with a yield strength of 235 MPa. The contact between the embedded bar bolt, steel bar, and concrete is calculated by applying the Lagrange algorithm defined by the lsdyna keyword of *CONSTRAINED_LAGRANGE_IN_SOLID. The single-surface contact is defined by *CONTACT_AUTOMATIC_SINGLE_SURFACE for the upper structure and lower barrier. The surface-to-surface contact between the barrier and vehicle is defined by *CONTACT_AUTOMATIC_SURFACE_TO_SURFACE. Constrain the 6 degrees of freedom of the end nodes of barriers and bridge decks in the length direction to simulate infinite length boundary conditions, which is defined by * BOUNDARY_SPC, that insert 1for translational constraint in x–y–z-direction and rotational constraint about x–y–z-axis. The established finite element model is shown in Fig. 11.

**Figure 12:**
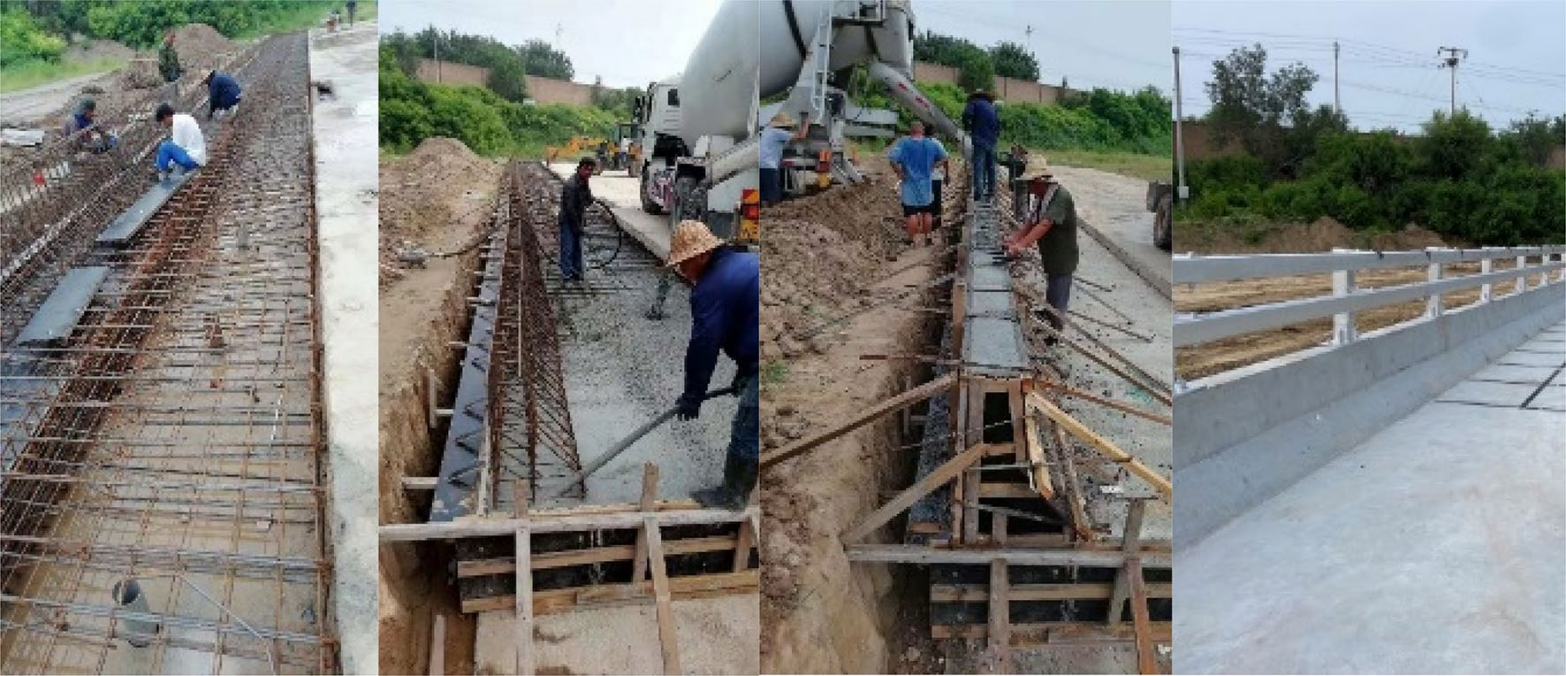
Test barriers.

As shown in Fig. 12, the universal double-beam assembled bridge barriers are constructed by binding steel bars around the formwork, pouring the bridge deck, pouring the concrete barrier base, and planting the steel bars. The strength test value of the “base concrete” in the guardrail test section for collision testing is 16.5 MPa; the strength test value of the guardrail test section “bridge deck” used for collision testing is 39.7 MPa. The performance parameters of guardrail materials is shown in Table 4.

**Table 4:** Performance parameters of guardrail materials.

Parameters	Field test values	Numerical-vehicle	Real-vehicle
Base concrete (MPa)	18	18	16.5
Bridge deck (MPa)	/	40	39.7
Yield strength of beam and post (MPa)	/	355	355

### FE analysis and Full-scale vehicle impact test verification

SS-level vehicle impact tests are conducted in accordance with *JTG B05-01-2013* to verify the performance of the improved universal double-beam assembled bridge barrier.

**Figure 13:**
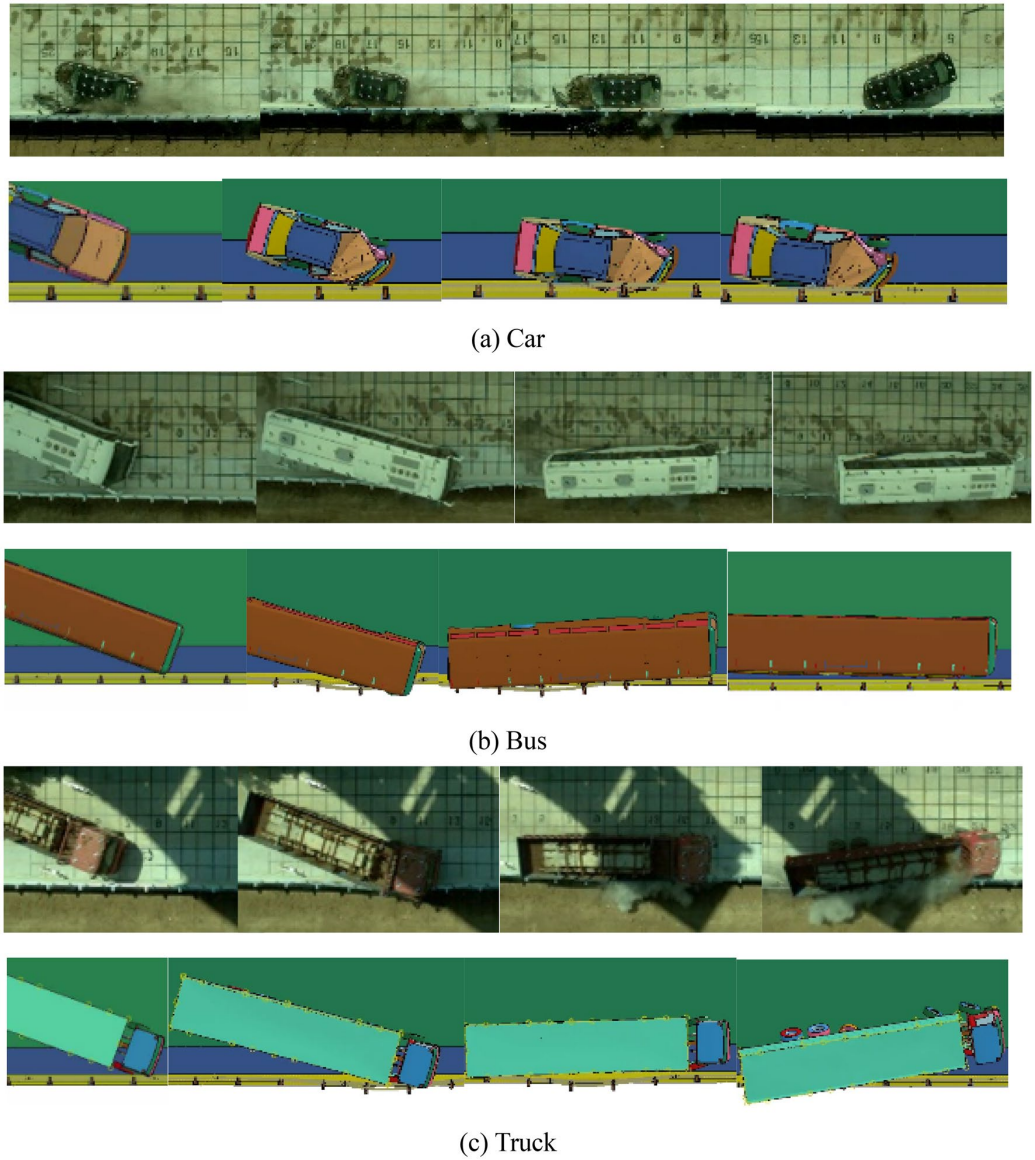
Comparisons of FE analysis and full-scale vehicle impact (from left to right).

For every type of vehicle, there are two rows of screenshots which are taken shot in 0.2 s respectively from left to right. Obviously, the car, bus, and truck all smoothly leave the impact zone after the impact, without crossing, climbing up, or riding over the barrier, as shown in Fig. 13. Thus, the containment performance complies with the requirements. And it is true that the travelling track of FE analysis result and full-scale vehicle impact record are nearly the same, which indicates the consistence of simulation and real test in this paper.

**Figure 14:**
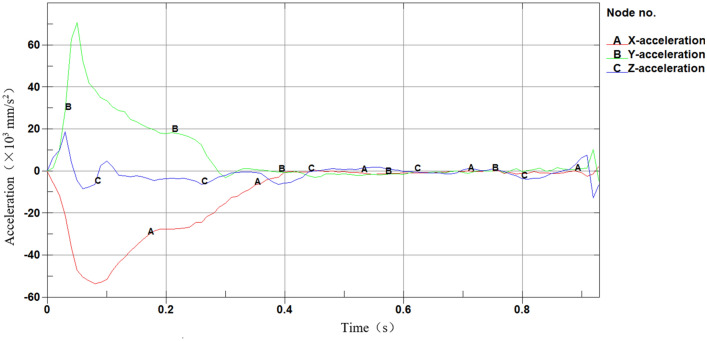
Acceleration of a car.

The centroid acceleration of the car is extracted. Define the X direction as the initial speed direction of the vehicle, and the Y direction as its vertical direction. The maximum acceleration is on the Y direction with a peak value of 70.9 m/s^2^, indicating that the barrier’s buffering performance meets relevant requirements (as shown in Fig. 14).

**Figure 15:**
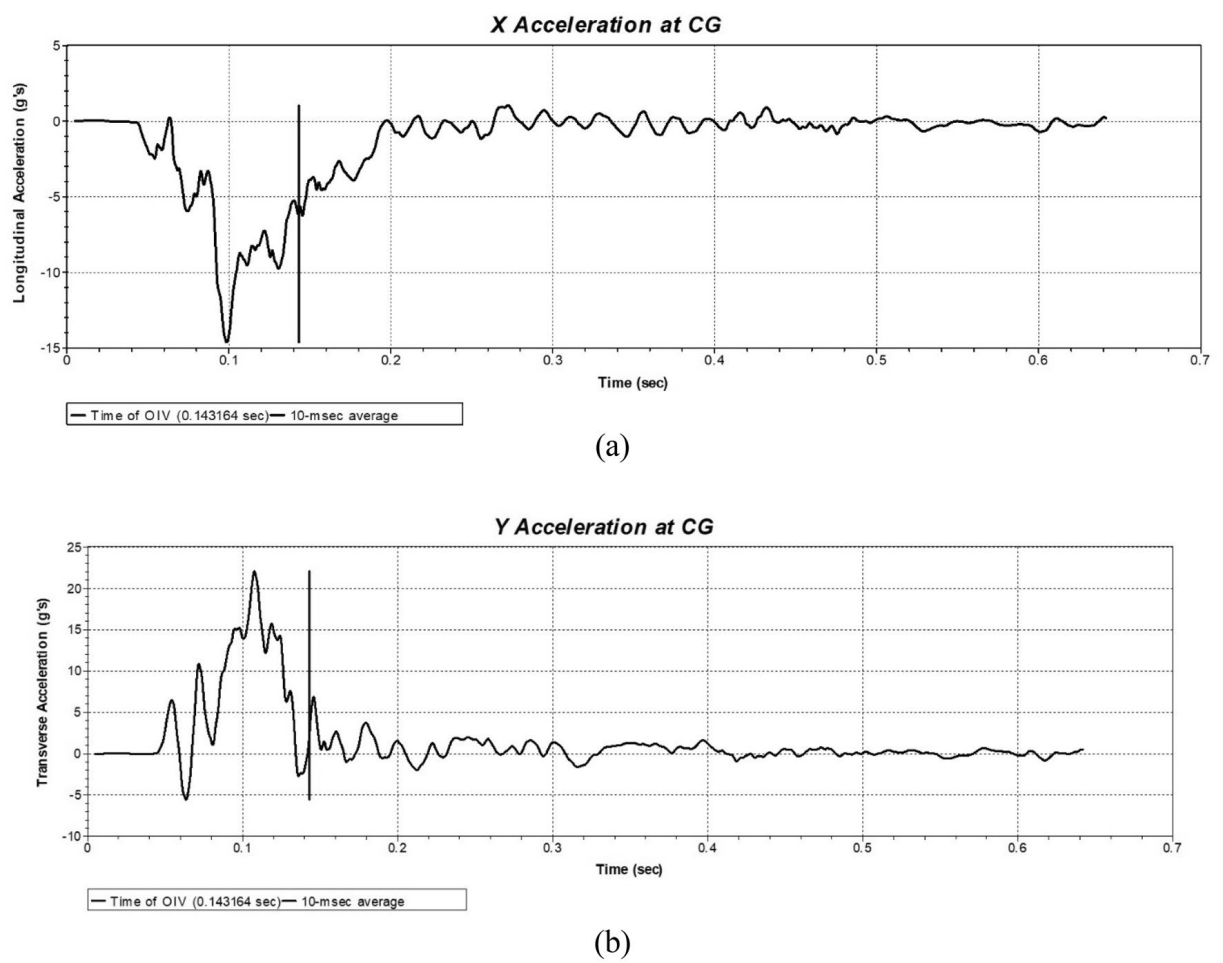
Time history curves of centroid acceleration of a car after collision.

The actual vehicle collision test tested the acceleration at the center of mass of the car. Using data processing software EXCEL, the acceleration curve is drawn by filtering at a frequency of 60 Hz and taking one test value every 9 values, so as to achieve an average value of 10 ms. The time-history curves of the longitudinal and transverse components of centroid acceleration of a car after the impact are shown in Fig. 15. When value of Y which represent the transverse displacement of driver is equal to 0.6 m, it means that the driver is likely to collide with car window. The larger of the acceleration of this moment is, the more danger the drive is. Displacement is calculated by integrating acceleration. When transverse displacement is 0.6 m, the maximum absolute values of the 10 ms interval average values of the longitudinal and transverse acceleration components are a_x_ = 45.08 m/s^2^ and a_y_ = 36.26 m/s^2^, respectively, both far less than 200 m/s^2^, meeting the evaluation standards.

According to the experimental data, the absolute values of the longitudinal and transverse components of the vehicle after impacting the barrier are calculated as v_x_ = 6.0 m/s and v_y_ = 7.3 m/s, respectively, less than 12 m/s, which shows that the occupant impact velocities is meeting the relevant requirements. The results indicate that the barrier’s buffering effect complies with the standards.

**Figure 16:**
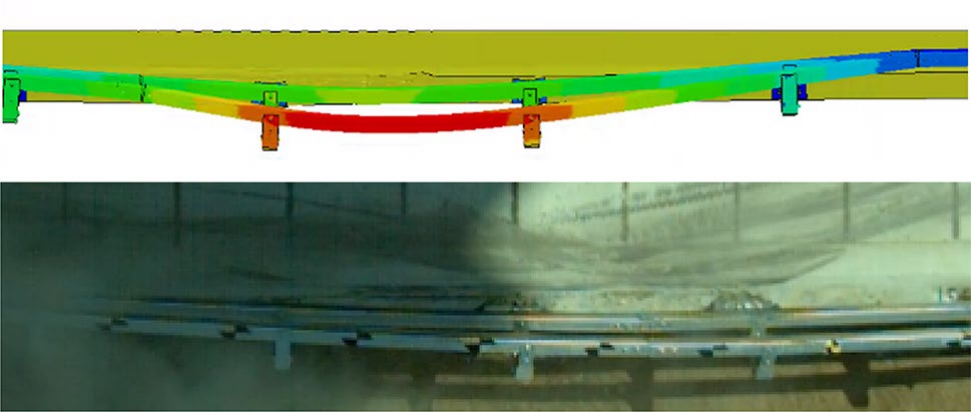
Barrier deformation after impacting truck.

The barrier’s deformation after impacting a truck is analyzed, the maximum dynamic deformation is 581 mm, and is 690 mm in the full-scale test. The difference in guardrail deformation values between computer simulation analysis and actual vehicle collision tests is only 10 cm, and the calculation results are close, as shown in Fig. 16.

Analyze the state of guardrails after different vehicles collide with them. After the impact of a small passenger car and the simulation collision test, there is a small area of concrete damage to the guardrail base. And, in the full-scale collision test of the actual vehicle, there are little obvious signs of damage to the guardrail base and crossbeam. This indicates that computer simulation tests can effectively improve the likelihood of passing real vehicle collision tests.

**Figure 17:**
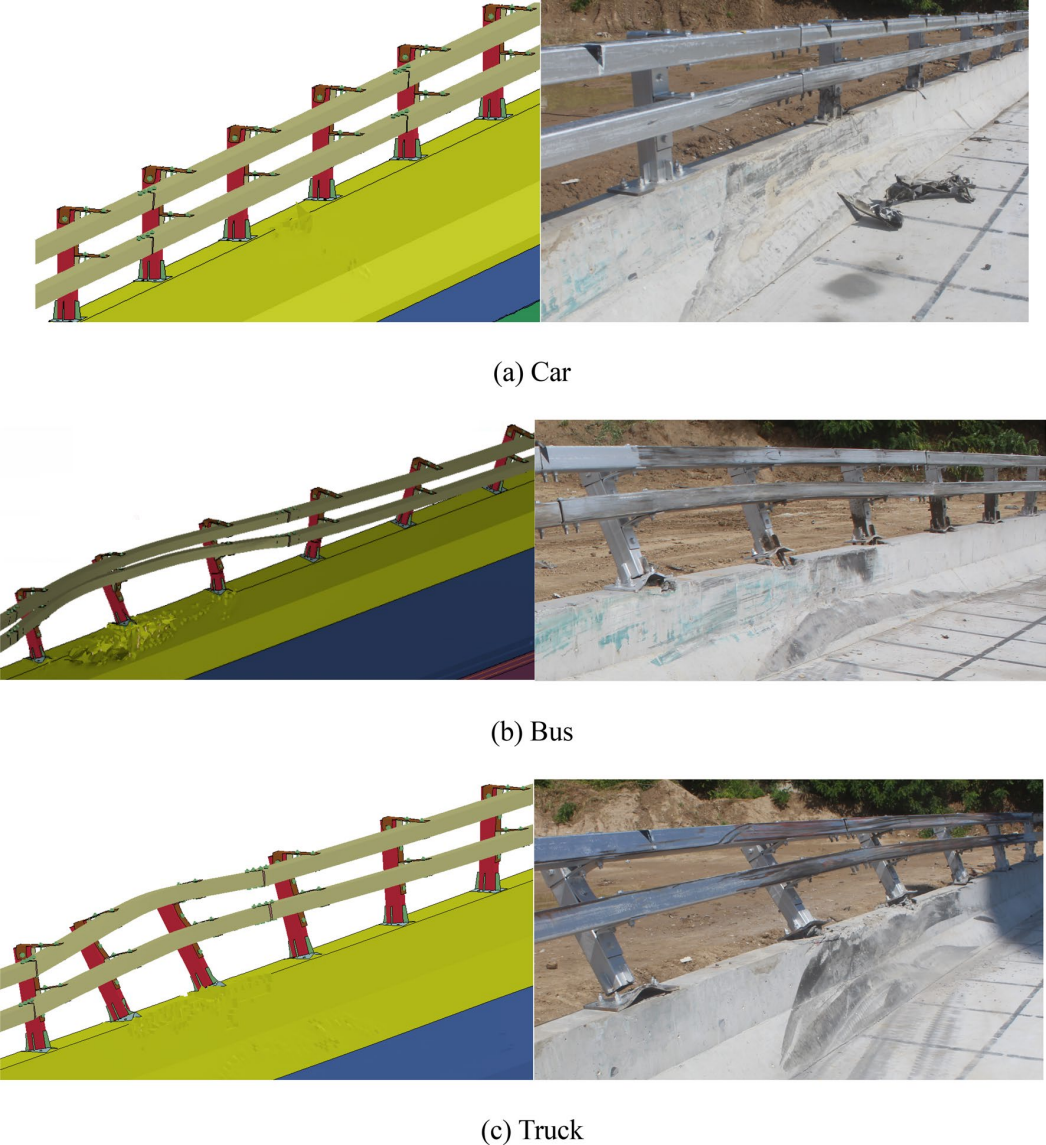
Barrier form after collision.

After the medium-sized bus collided with the guardrail, in the simulation test, the large deformation length of the crossbeam is 6 m, and the guardrail base is damaged within a range of 6 m. In the actual vehicle collision test, four flange columns are damaged, and the concrete base is damaged with a length of 6 m; After a large truck collided with a guardrail, in the simulation test, the large deformation length of the crossbeam is 6 m, and the damaged length of the concrete base is 6 m, and this is also true in actual vehicle collisions, as shown in Fig. 17.

**Figure 18:**
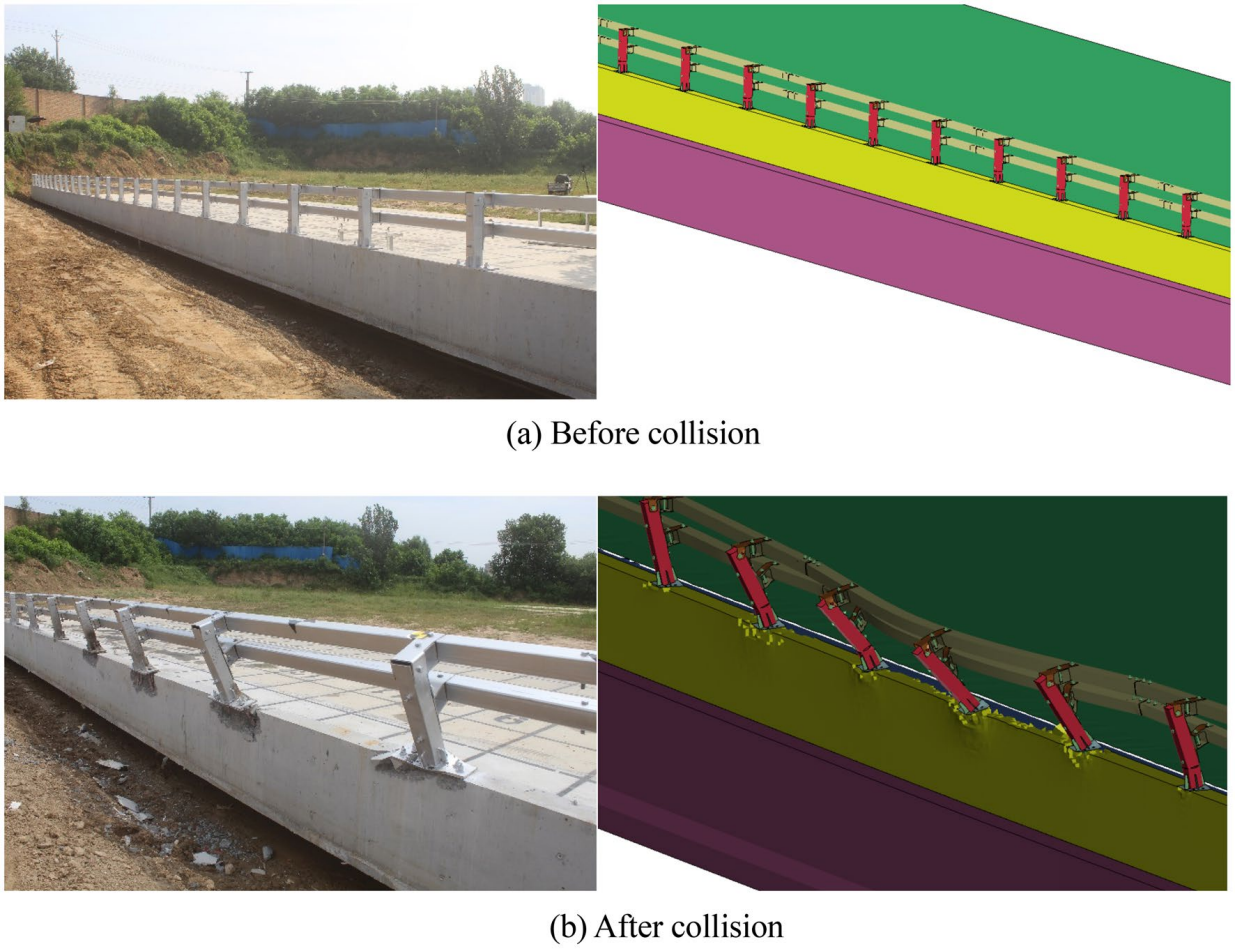
Barrier form after impacting.

After the impact of a medium-sized bus, the barrier concrete is most severely damaged. Therefore, the damage status of the bridge deck after a medium-sized bus collides with the barrier is analyzed. As shown in Fig. 18, after the medium-sized bus collided with the guardrail, the damage marks on the guardrail concrete did not extend to the bridge deck, the bridge deck was intact and undamaged, and the connection between the guardrail and the bridge deck was firmly connected in the simulation test and the actual vehicle collisions.

According to the simulation results and the real-vehicle impact test results, the universal double-beam assembled barrier meets all SS-level requirements in terms of containment, redirective and buffering, the universal double-beam assembled bridge barrier can effectively handle the impact of car, bus and truck, and the overall safety performance reaches Level VI (SS).

## Conclusions

1. In this paper, the worst-case concrete barrier base and bridge deck of a highway are determined. A scheme to improve the safety performance of bridge barrier with the existing concrete base and a universal double-beam assembled bridge barrier is developed. The new configuration can effectively improve the protection ability of different types of barriers and deck slabs on the same expressway.
2. SS-level real-car impact tests are conducted to small and buss, and trucks. The results show that the universal double-beam assembled bridge barrier has achieved SS-level in protection capability and can be universally used for improving barriers with a concrete base at a height of 63 cm and above.

## Data Availability

The data support the findings of this study are available from the corresponding author upon reasonable request.
